# The role of macrophage IL-10/innate IFN interplay during virus-induced asthma

**DOI:** 10.1002/rmv.1817

**Published:** 2014-11-27

**Authors:** Mihnea T Zdrenghea, Heidi Makrinioti, Adriana Muresan, Sebastian L Johnston, Luminita A Stanciu

**Affiliations:** 1 Ion Chiricuta Oncology InstituteCluj-Napoca, Romania; 2 Iuliu Hatieganu, University of Medicine and PharmacyCluj-Napoca, Romania; 3 Airways Disease Infection Section, National Heart and Lung Institute, Imperial College LondonLondon, UK; 4 Medical Research Council and Asthma UK Centre in Allergic Mechanisms of AsthmaLondon, UK; 5 Centre for Respiratory InfectionsLondon, UK

## Abstract

Activation through different signaling pathways results in two functionally different types of macrophages, the pro-inflammatory (M1) and the anti-inflammatory (M2). The polarization of macrophages toward the pro-inflammatory M1 phenotype is considered to be critical for efficient antiviral immune responses in the lung.

Among the various cell types that are present in the asthmatic airways, macrophages have emerged as significant participants in disease pathogenesis, because of their activation during both the inflammatory and resolution phases, with an impact on disease progression. Polarized M1 and M2 macrophages are able to reversibly undergo functional redifferentiation into anti-inflammatory or pro-inflammatory macrophages, respectively, and therefore, macrophages mediate both processes.

Recent studies have indicated a predominance of M2 macrophages in asthmatic airways. During a virus infection, it is likely that M2 macrophages would secrete higher amounts of the suppressor cytokine IL-10, and less innate IFNs. However, the interactions between IL-10 and innate IFNs during virus-induced exacerbations of asthma have not been well studied.

The possible role of IL-10 as a therapy in allergic asthma has already been suggested, but the divergent roles of this suppressor molecule in the antiviral immune response raise concerns. This review attempts to shed light on macrophage IL-10–IFNs interactions and discusses the role of IL-10 in virus-induced asthma exacerbations. Whereas IL-10 is important in terminating pro-inflammatory and antiviral immune responses, the presence of this immune regulatory cytokine at the beginning of virus infection could impair the response to viruses and play a role in virus-induced asthma exacerbations.

## Introduction

The greatest burden of asthma, a chronic inflammatory lung disease that affects all age groups, lies in acute exacerbations that require intense treatment and possible hospitalization [1,2]. Respiratory viruses are the major triggers of asthma exacerbations [3,4]. The production in the lung of antiviral innate IFNs and immunosuppressor cytokine IL-10 plays an important role in virus-induced asthma exacerbations, but their interactions are poorly understood.

Macrophages (MØs) have emerged as significant participants in virus-induced asthma pathogenesis, mainly because of their activation during both the inflammatory and resolution phases, with an impact on disease progression. MØs have both antigen-presenting and regulatory functions, and they are also highly plastic.

A balanced polarization of MØs toward M1 or M2 is considered critical in efficient antiviral immune responses in the lung and plays a decisive role during virus-induced exacerbations of asthma. M2 MØs produce IL-10 that can signal via its receptor on responding/target cells and thereby decrease the secretion of soluble factors such as antiviral innate IFNs. The possible role of IL-10 as a therapy in allergic asthma has already been suggested [5]. However, the divergent roles of this suppressor molecule in immune responses raise concerns. This review attempts to shed light on MØ IL-10 and innate IFN interactions and discusses the role of IL-10 in virus-induced asthma exacerbations.

## Macrophage Phenotypes

**Figure 1:**
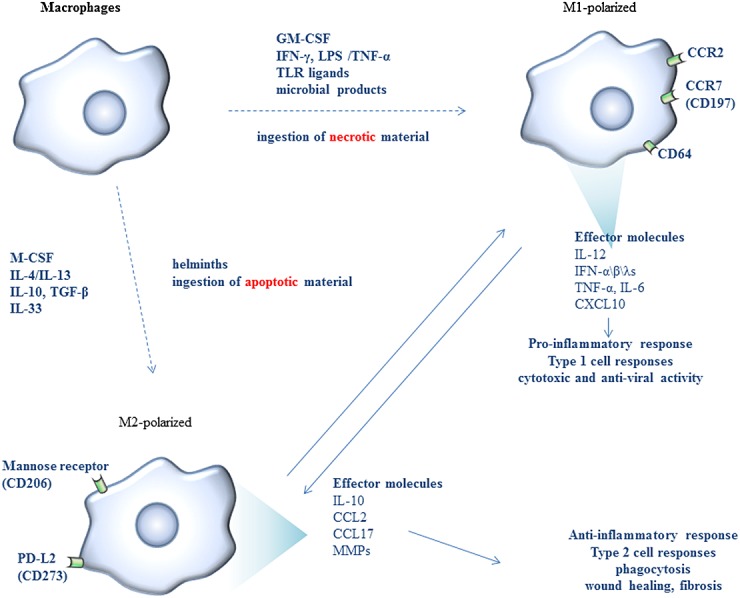
Macrophage differentiation pathways. Activation through different signaling pathways results mainly in two types of macrophages, the classically activated (M1) and the alternatively activated (M2), which can reversibly undergo functional redifferentiation upon environmental changes. Functionally, M1 macrophages show increased cytotoxic and antiviral activity, while M2 macrophages help tissue homeostasis to be restored by inducing wound healing, angiogenesis, and fibrosis

Activation through different signaling pathways results in two functionally different types of MØs, the inflammatory MØs (or classically activated MØs (caMØ), or M1) and the anti-inflammatory (or non-classically, alternatively activated MØs (aaMØ) or M2) (Figure1 and Table1).

**Table 1:** Representative studies describing the different phenotypes of macrophages

Ref	Outcome	Mechanism	Model	*p*
[9]	IRF5 upregulates the expression of established phenotype of M1 and downregulates the expression of M2 macrophages.	The secretion of IL-12p70, IL-23, IL-12 p40, and IL-10 by M2 macrophages infected with adenoviral vector encoding IRF5 was much greater than the secretion of empty vector (pENTR) infected.	*In vitro*	<0.05
				<0.01
				<0.001
		mRNA expression of ^*^^*^IL-12p40, ^*^IL-12p35 and ^*^IL-23p19, and ^*^^*^^*^IL-10 in M2 macrophages infected with adenoviral vector encoding IRF5 or IRF3 is relatively similar to the control cells infected with empty vector.	*Ex vitro*	<0.05(^*^)
				<0.01(^*^^*^)
				<0.001(^*^^*^^*^)
		Treatment of monocytes with GM-CSF resulted in an increased IRF5 mRNA expression within 2 h of stimulation, as opposed to treatment with M-CSF.	*Ex vivo*	<0.001
[13]	Iron induces an unrestrained M1 macrophage population that expressed both classical M1 markers (TNF-α, iNOS, IL-12, and CCR2) and M2 markers, particularly the scavenger receptors CD163 and CD206, and exhibited low expression of IL-4Rα, IL-10, Dectin-1, CD36, arginase, M2 receptors, and cytokines.	Macrophages isolated from wound margins of iron-dextran-treated mice revealed an activation pattern reminiscent of macrophages isolated from chronic venous leg ulcers (CVUs) with a persistent pro-inflammatory M1 response and intermediate anti-inflammatory M2 marker activation.	*Ex vivo*	<0.01
		A chimeric IgG1 monoclonal antibody that binds with high affinity and specificity to the soluble and transmembrane TNF-α resulted in significant improvement of healing in 12 of 14 previously therapy-resistant CVUs.	*Ex vivo*	<0.01
[14]	Cytokines can favor alternate activation of macrophages with a high phagocytic capacity toward infected cells.	Macrophages differentiated in the presence of M-CSF showed a twofold to threefold greater phagocytic capacity compared with GM-CSF-induced cells.	*Ex vivo*	<0.05
		The addition of IL-10 significantly increased, whereas IL-4 decreased phagocytosis by both M-CSF-differentiated and GM-CSF-differentiated macrophages.	*Ex vivo*	<0.01
[17]	Macrophages sequentially change their functional phenotype in response to changes in microenvironmental influences. Therefore, sequential treatment of macrophages with multiple cytokines results in a progression through multiple phenotypes.	Macrophages display distinct functional patterns after treatment with IFN-γ, IL-12, IL-4, or IL-10.	*Ex vivo*	<0.05
		Additional functional patterns are displayed depending on whether the cytokine is present alone or with other cytokines and whether the cytokines are added before or concomitantly with the activating stimulus.	*Ex vivo*	<0.05

M1 MØ polarization is induced by IFN-γ, lipopolysaccharide (LPS) and/or exposure to TNF-α or granulocyte MØ colony-stimulating factor (GM-CSF), or other microbial products and ingestion of necrotic material. M1 MØs have been shown to act as efficient antigen-presenting cells, and they also have increased cytotoxic and antiviral activity [6,7]. They play an important role in defense against viruses, bacteria, and protozoa and drive pro-inflammatory responses.

The M2 MØ phenotype is induced by IL-4, IL-13, IL-10, IL-33, transforming growth factor-beta (TGF-β), and the ingestion of apoptotic material. M2 differentiation has been shown to be mediated via signal transducer and activator of transcription (STAT)3 and STAT6 pathways, while STAT1 and nuclear factor-kappa B (NF-κB) pathways are implicated in M1 polarization [8,9]. Among all the aforementioned stimulants, IL-4 has been considered as the most powerful inducer of the M2-phenotype in alveolar MØs and monocytes [10,11]. M2 MØs play a role in defense against helminths and in allergic responses [7]. Analysis of the gene expression profile in GM-CSF-polarized and M-CSF-polarized MØs revealed that a high CCL2 expression characterizes MØs generated under the influence of M-CSF, whereas CCR2 is expressed only by GM-CSF-polarized MØs.

M2 MØs produce high levels of IL-10, low levels of IL-12, and Th2-attracting chemokines (MØ-derived chemokine/chemokine ligand 22 or MDC/CCL22, thymus and activation-regulated chemokine/chemokine ligand 17 or TARC/CCL17, and pulmonary and activation-regulated chemokine/chemokine ligand 18 or PARC/CCL18), resistin-like molecule-α (RELMα or FIZZ1), chitinase-like proteins, and arginase 1 (ARG1). M2 have a decreased ability to kill pathogens such as bacteria and viruses and decrease the magnitude and duration of inflammatory responses by antagonizing M1-mediated and type 1 T cell-mediated responses mainly by an increased anti-inflammatory IL-10 cytokine production [12,13].

M2 MØs show a poor antigen-presenting capability, but they express receptors involved in phagocytosis, in the engulfment and digestion of dead cells, debris, and various extracellular matrix components that could promote tissue-damaging M1 MØ responses [8,14]. More interestingly, M2 MØs produce factors that induce the apoptosis of myofibroblasts, as well as matrix metalloproteinases and tissue inhibitors of metalloproteinases (TIMPs) that control extracellular matrix turnover and play an important role in wound healing, angiogenesis, and fibrosis by helping tissue homeostasis to be restored [15].

A subdivision of M2 MØs was recently proposed: (i) M2a (differentiated with M-CSF and stimulated with IL-4 and IL-13); (ii) M2b (or type 2-activated, MØ-II, or regulatory, following stimulation by immune complexes in the presence of a Toll-like receptor (TLR) ligand); (iii) immunoregulatory M2c (when exposed to anti-inflammatory stimuli such as glucocorticoid hormones, IL-10, or TGF-β); and (iv) M2d (driven by a tumor microenvironment). All M2 subpopulations have been associated with increased IL-10 and reduced inflammatory responses [16].

### Polarized macrophages have the ability to repolarize

Polarized pro-inflammatory M1 and anti-inflammatory M2 MØs are not at an end stage of differentiation but are able to reversibly undergo functional redifferentiation into anti-inflammatory or pro-inflammatory MØs upon environmental changes [17–20].

*In vitro*, M1 and alternatively activated M2a monocyte-derived MØs (MDMs) have shown a complete reversion to control levels 1 week after the removal of polarizing cytokines, indicating significant plasticity [19]. Also, GM-CSF-driven M1 MØs, when exposed to M-CSF for less than 1 week, have been shown to acquire an M2-like phenotype, with reduced production of the pro-inflammatory cytokines IL-6 and TNF-α and reduced capacity of stimulating T cells [20]. Conversely, it has been reported that M2 MØs tend to express M1-associated genes upon exposure to TLR ligands or IFN-γ [17,18].

Macrophage phenotype is equally flexible *in vivo*. Typical M1 and M2 phenotypes are extremes of a spectrum of functional states with intermediate or overlapping phenotypes observed in infections [21]. Although most types 1 and 2 cytokines have not been able to induce a stable differentiation of MØs into distinct subsets, recent studies have shown that basophil-derived IL-4 can trigger M1-polarized MØs to “convert” into anti-inflammatory MØs with an M2 wound-healing phenotype [22].

Although it has been suggested that M2 activation happens *in situ* and not systemically and that local tissue MØs undergo proliferation in type 2-mediated inflammation, it is still unclear whether in type 2 cytokine-dominated diseases such as allergic asthma, this differentiation occurs systemically as well. Further studies are required in order to shed light on and identify whether local treatment would therefore be considered as a solution [23–26].

### IL-10 production by monocytes/macrophages

Alternatively activated MØs produce high levels of IL-10, which can concomitantly signal through its receptor in an autocrine manner in MØs and/or in a paracrine manner in surrounding cells, modulating the expression and secretion of soluble factors and surface molecules.

The IL-10 activity is mediated by interaction with its own receptor, IL-10 hetero-dimeric receptor (IL-10R), composed of two chains IL-10R1 (IL-10Rα) and IL-10R2 (IL-10Rβ). It has been shown that IL-10 initially binds to its signaling receptor IL-10R1 (IL-10Rα) and that this interaction changes the cytokine conformation by allowing the association of the IL-10–IL-10R1 (IL-10Rα) complex with IL-10R2 (IL-10Rβ) [27]. Monocytes and MØs exhibit the highest IL-10R expression [28]. IL-10 confers broad anti-inflammatory responses in IL-10R1 (IL10Rα)-expressing cells, and it has been proposed that these responses are amplified in a feed-forward mechanism encompassing STAT3-dependent transcriptional induction of IL-10 [29].

In contrast to IL-10R1, IL-10R2 is widely and strongly expressed in most cells and tissues that do not express IL-10R1 [30], and IFN-γ or TNF-α treatment increases IL-10R2 mRNA expression [31,32]. However, IL-10R2 alone is unable to bind IL-10 [33], and IL-10R2 is the common subunit for other receptors recognizing other cytokines [34].

Monocytes/MØs are the main target cells of the inhibitory effects of IL-10. It has been reported that IL-10, through interaction with its own receptor, decreases the antigen-presenting function and pro-inflammatory cytokine production, as well as the microbicidal activity of monocytes/MØs [35,36]. *In vivo* studies have shown that IL-10 regulates its own production by monocytes/MØs in an autocrine manner via activation of the transcription factor STAT3 [37]. STAT3 activates the suppressor of cytokine signaling 3 (SOCS3), which controls the quality and quantity of STAT activation [38]. SOCS3 is induced by IL-10 and exerts negative regulatory effects on various cytokine genes. There is no evidence though that IL-10R has SOCS-binding sites, and therefore, it has not been shown that IL-10R is subject to regulation by SOCS3 [39,38].

Of increased interest is the interaction between IL-10-producing MØs and regulatory T cells. IL-10 produced by MØs induces regulatory T cells and creates an immunosuppressive environment [29]. Regulatory T cells induce alternative activation of human monocytes/MØs via IL-10, IL-4, and IL-13 and other cytokine-independent pathway(s) [40]. IL-10, but not IL-4/IL-13, has been shown to upregulate the expression of M2 surface marker CD163, type 2 chemokine PARC/CCL18, and phagocytosis [40]. In addition, IL-10 may act directly and suppress the function of CD4 T cells [41] and memory CD8 T cells [42].

### Antiviral innate IFN production by monocytes/macrophages

Type I IFNs—IFN-α subtypes and IFN-β—are a group of cytokines with antiviral, pro-apoptotic, antiproliferative, antitumor, anti-angiogenic, and anti-inflammatory effects. The type I IFN receptor (IFNR) complex consists of two transmembrane chains: the IFN-α receptor (IFNAR)-1 and IFNAR-2 chains. Three different forms of IFN-αR2 mRNA have been reported (IFN-αR2a, IFN-αR2b, and IFN-αR2c mRNA), but only the IFN-αR2c protein mediates a biologic response when associated with the IFN-αR1 protein [43]. Among the 17 human IFN subtypes, IFN-β binds the IFNAR-1 and IFNAR-2 chains with particularly high affinity and is especially potent in select bioactivities (e.g., antiproliferative and pro-apoptotic) when compared with IFN-α2 [44].

Macrophages are less permissive to viral replication in comparison with epithelial cells, possibly because of a low-grade “spontaneous” production of type I IFNs (such as IFN-α and IFN-β) produced by uninfected cells [45,46]. The intrinsic antiviral activity depends among other factors on MØ differentiation and has been correlated to IFN activity, as defined by physiological levels of constitutive pre-infection production or by rapidly acting autocrine IFN-α/β [47]. NF-κB subunit p65 (RelA) sustains autocrine IFN-β signaling prior to infection in uninfected cells [48], and NF-κB and AP-1 subunit c-Jun sustain basal/early IFN-β expression [49]. Three transcription factors, IFN regulatory factor (IRF)-3, IRF-7, and IFN-stimulated gene factor 3 (ISGF3) (a complex consisting of phospho-STAT1, phospho-STAT2, and IRF-9), are crucial in regulation of type I IFNs following virus infection IFNs [49,50]; IRF-3 is a constitutively expressed protein in most cell types that shuttles between the cytoplasm and nucleus. Once IRF-3 is phosphorylated at its C-terminus, it remains localized in the nucleus where it serves as a transcription factor, in conjunction with AP-1 and NF-κB, inducing IFN-β and IFN-α1 transcription [51,52].

Type III IFNs (IFN-λ1/2/3 also referred to as IL-29/IL-28A/IL-28B) are related to both type I IFNs and IL-10 and have antiviral activity and signal through a hetero-dimeric receptor composed of IFN-λR1/IL-28Rα and IL-10Rβ/IL-10R2 chains. IL-29/IFN-λ1 initially binds to signaling receptor IFN-λR1/IL-28Rα and causes a conformational change that subsequently allows the IL-10Rβ/IL-10R2 receptor to bind to IL-29/IFN-λ1. The receptor complex is activated and induces IFN-stimulated genes, which inhibits viral replication by interfering with viral RNA transcription and protein translation, but also has immunostimulatory and antiproliferative effects [53]. The IFN-λ1/IL-29 gene is regulated by virus-activated IRF-3 and NF-κB, resembling that of the IFN-β gene, whereas IFN-λ2 and/or IFN-λ3 gene expression is mainly controlled by the IFN-stimulated gene IRF-7, resembling regulation of IFN-α genes and suggesting that IFN-λ2/IFN-λ3 could be expressed without IFN-λ1 [54–56].

The precise interactions between type I and type III IFNs have not been well defined. It has been reported that IFN-α amplifies the induction of IFN-λ expression by influenza or Sendai virus [57,58] partially by upregulating TLR and IRF-7 gene expression [58,59]. However, recent studies have shown that the IFN-α and IFN-λ ligand–receptor systems can be activated independently in response to certain viruses and that type I IFN receptor IFNRA signaling is not essential for IFN-λ production in epithelial cells [60,61].

It was suggested that IFN-λ expression is more flexible than IFN-α/β expression, which could allow expression of type III IFNs in response to a wider range of stimuli compared with type I IFNs and will potentially make the expression of type III IFNs less sensitive to microbial evasion strategies targeting the IRF pathway [62,63]. In the absence of IRF-3 activation and IFN-β production, alternative pathways allow IFN-λ induction in the absence of IRF-3 activation [64].

The p50 NF-κB homodimer was identified as a key repressor of the IFN-λ1 gene and a key regulator of M2-driven inflammatory reactions *in vitro* and *in vivo*: p50 NF-κB inhibits NF-*κ*B-driven M1 polarization, IFN-β production [65,66], and IFN-λ1 gene expression [67].

## Type III IFNs Share the IL-10RΒ/IL-10R2 with IL-10 but have Functions Similar to Type I IFN

Type III IFNs are related to both type I IFNs and IL-10. The similarity in structure between IFN-λ and members of the IL-10 family probably reflects a common evolutionary origin as well as the evolutionary restraint caused by sharing a common receptor chain [53].

The receptor of IL-29/IFN-λ1 is expressed by a number of cell types, including freshly isolated PBMCs, MØs, plasmacytoid dendritic cells (pDC), B cells, T cells, epithelial cells, and hepatocytes [60,68–70]. Monocytes and MDMs express low levels of signaling IFN-λR1/IL-28Rα receptor and high levels of IL-10R1, IL-10R2, IFN-αR2c, and IFN-αR1 [70–73].

Despite the similarities of the IFN-λ receptor complex to the IL-10 receptor complex, the signal transduction pathways for IFN-λ are very different and almost identical to the ones induced by type I IFNs [74–76], inducing nearly identical patterns of gene expression and consequently similar induction of antiviral and antiproliferative activity [77,78]. Similar to type I IFNs, type III IFNs downregulate type 2 cytokines (IL-13, IL-4, and IL-5), inhibit GATA-binding protein 3 (GATA3) expression, and suppress type 2 immune responses, while they trigger a more modest elevation of IFN-γ secretion (a Th1 response) [79–81]. Which mechanisms are involved is not well known.

An antagonistic role for IL-10 and type III IFNs has been suggested. Jordan *et al*. speculated that IL-10 may act as an antagonist to IL-29/IFN-λ1 functions, signifying a highly sensitive IL-10-dependent feedback mechanism that regulates the function of IL-29/IFN-λ1 and/or that IL-10 and IFN-λ compete for the IL-10R2/IL-10Rβ chain in their respective receptors [82]. Understanding the mechanisms by which innate IFNs and IL-10 interact will provide important information for the identification of their role in virus-induced asthma.

Recently, differential regulation of IFN-γ receptor 1 chain by IL-29 (but not IL-28A or IL-28B) and IFNα/β on myeloid cells was reported. IL-29 pretreatment upregulated the IFN-γR1 chain, increasing MDM response to IFN-γ stimulation and inducing high levels of IL-12p40 in response to R848, whereas IFN-α downregulated IFN-γR1 expression and suppressed IFN-γ–induced IL-12p40 by MDM on TLR7 ligation [73]. Another group reported that IFNα/β downregulation of IFN-γR1 expression by MØs correlated with reduced responsiveness to the pro-inflammatory cytokine IFN-γ and increased susceptibility to bacterial infections [83].

## Viruses Increase Innate IFN Production in Monocytes/Macrophages

Hillyer *et al*. reported that both the stimulant and the cell type determined consequent human innate IFN expression patterns [84]. In response to poly-I:C, a dsRNA ligand for TLR3, monocytes and MDMs express a pattern restricted primarily to IFN-β and IFN-λ1 via TLR3/Toll/IL-1 receptor (TIR) domain-containing adaptor protein-inducing IFN-β (TRIF)/IRF-3 signaling. TLR3 recognizes extracellular and endosomal dsRNA, and rather than signaling through MyD88, it associates with TRIF to activate both NF-κB and IRF-3 [85,86]. MØs show increased expression of innate IFNs in response to virus signaling via TLR3 [57,84,87].

### Innate IFNs modulate IL-10 and pro-inflammatory cytokines

The interactions between innate IFNs, pro-inflammatory cytokines, and IL-10 during acute viral infections are not well defined, and current data are contradictory. The divergent data could be explained either by different stimulants that have been used or because the studies have been conducted *in vitro* or *in vivo*, in animals or in humans.

Studies using human monocytes found that type I IFNs inhibit pro-inflammatory IL-12 p40 production at the transcriptional level [88]. When fresh human blood cells from healthy subjects were treated with IFN-β and flow cytometry was used to analyze phosphorylated STAT levels, monocytes activated STAT1 in response to IFN-β-induced and IFN-β-induced STAT1-dependent pro-apoptotic mRNAs in monocytes [89]. *In vitro*, human MDMs when stimulated with IFN-α, IFN-β, and IL-29 upregulated the expression of the signaling receptor IL-10R1 (not IL-10R2) and triggered IL-10-induced STAT3 phosphorylation [90].

Data from animal studies suggest that type I IFNs increase the levels of anti-inflammatory IL-10 and decrease pro-inflammatory cytokines (IFN-γ, IL-12, and other type 1 cytokines) [91]. In a mouse model of *Pneumocystis* lung infection, in wild-type mice, the immediate progression to a Th2-mediated response was associated with an early induction of the immune regulatory cytokine IL-10 at Day 7, and mice exhibited minimal evidence for lung damage during the maximal immune response at Day 14, suggesting a protective role for IL-10 in resolving the immune responses [91,92]. However, the absence of IFNAR results in a delayed but exacerbated Th2-mediated immune response, followed by lung fibrosis by Day 35 postinfection despite pathogen clearance when compared with wild-type mice in which the inflammation results in complete restitution [91,92].

Type I IFNs are responsible not only for the direct resolution of influenza A viral infection but also for suppressing any immunopathology caused by viruses via IL-10 production by monocytes/MØs [93]. It has been clearly shown that IFNAR knockout mice exhibit increased mortality and morbidity with higher viral load and higher levels of pro-inflammatory cytokines detected in the lungs as well as with lower levels of IL-10 [93]. This interactive pathway is of increased translational value, as the efficacy of innate IFN-α therapy in chronic hepatitis C virus infection and of IFN-β therapy in multiple sclerosis is attributed mainly to ability of IFNs to increase IL-10 production from innate immune cells important for its anti-inflammatory properties [94,95].

## The Role of Macrophage IL-10 Production in the Host Response to Acute Respiratory Virus Infection

Alveolar MØs, together with epithelial cells, act as the first-line sensor of invading viruses in the lung, responding by innate IFN and pro-inflammatory mediator production. Innate IFNs produced by MØs recognize invading viruses and activate surrounding cells, in an autocrine/paracrine manner, in order to increase resistance to the virus infection and remove virus-infected cells [96]. MØs produce IL-10 later on, which then participates in a feedback inhibitory loop, suppressing activation of MØs and inflammatory cytokine production at sites of damage and thus limiting toxicity and tissue damage [97,98].

The timing of IL-10 expression and production by MØs during a virus infection could be a particularly important feature. Decreased IL-10 activity in the acute phase of infection has been suggested to be beneficial to the host as it triggers enhanced immunity and clearance of the pathogen [99]. *In vitro* exposure to IL-10 selectively suppressed natural killer (NK) cell IFN-γ production [100], and the induction of IL-10, rather than type I IFNs, is accompanied by a temporary attenuation of NK and T-cell responses in the early stages of acute HBV infection [101]. The blockade of IL-10 signaling increased virus-specific CD8 and CD4 T cells and enhanced their function, resulting in the resolution of chronic viral infection [102,103].

During respiratory viral infections (influenza, respiratory syncytial virus, and rhinoviruses (RVs)), animal models suggest that early upregulated IL-10 levels at the beginning of a viral infection can be correlated with high virus infiltration, while the presence of IL-10 later on during the viral infection may prove beneficial and may play an important role in resolving the inflammatory responses [104]. RSV infection could trigger an excessive IL-10 response leading to downregulation of antiviral defense mechanisms and reduced elimination of respiratory pathogens. In RSV-infected children, IL-10-production by PBMCs correlated with the development of recurrent wheezing later on [105,106], and levels were increased in cases of RSV bronchiolitis [107]. In addition, IL-10 levels during RSV infection in nasopharyngeal aspirates were higher in infants that later developed physician-diagnosed postbronchiolitis wheeze as compared with infants without postbronchiolitis wheeze in the first year after RSV infection [108]. These clinical studies in children suggest that increased levels of IL-10 by RSV infection correlate with severe lung diseases.

### Alveolar macrophage phenotype and IL-10 in atopic asthma

Because of its immunosuppressive and anti-inflammatory properties, it has been suggested that IL-10 and regulatory T cells could be of therapeutic benefit in the treatment of allergic diseases such as atopic asthma [109–111]. However, there are studies in mice and man suggesting that IL-10 may contribute to asthma pathogenesis.

In mouse asthma exacerbation models, IL-10 augments eosinophilic inflammation, airway hyperresponsiveness (AHR), mucus metaplasia, IL-5 production, and airway remodeling [112,113], and M2 phenotype MØs were suggested to contribute to the pathogenesis of disease [114–116]. Alveolar MØ from ovalbumin (OVA)-sensitized allergy-susceptible Brown Norway (BN) rats released more IL-10 than alveolar MØ from allergy-resistant Sprague Dawley (SD) rats 24 h after OVA challenge, and the transfer of alveolar MØ from SD rats to the BN rats suppressed the AHR [117].

In human asthma studies, there is evidence suggesting that the dominant MØ phenotype in atopic asthmatic subjects is alternatively activated (aaMØ or M2), characterized by increased production of IL-10. Increased IL-10 expression in sputum and increased IL-10 production in monocytes and alveolar MØ have been reported in asthmatics compared with controls [118–124]. Bronchoalveolar lavage (BAL) fluid levels of type 2 chemokines TARC/CCL17 and MDC/CCL22, most probably produced by alveolar aaMØ, were increased after segmental challenge and correlated with airway eosinophils and concentrations of IL-5 and IL-13 in allergic asthma [125,126], and higher numbers of arginase-1-positive cells, predominantly MØ, in BAL cells, were reported in allergic asthmatic as compared with control subjects [127].

### The role of the balance IL-10/innate IFN macrophage production upon rhinovirus infection in virus-induced asthma

The presence of the suppressor cytokine IL-10, produced by MØs during a virus infection, by downregulating mediators associated with type 1 antiviral immune responses, could delay virus eradication and even allow viral persistence [35,36,105,128–130].

**Figure 2:**
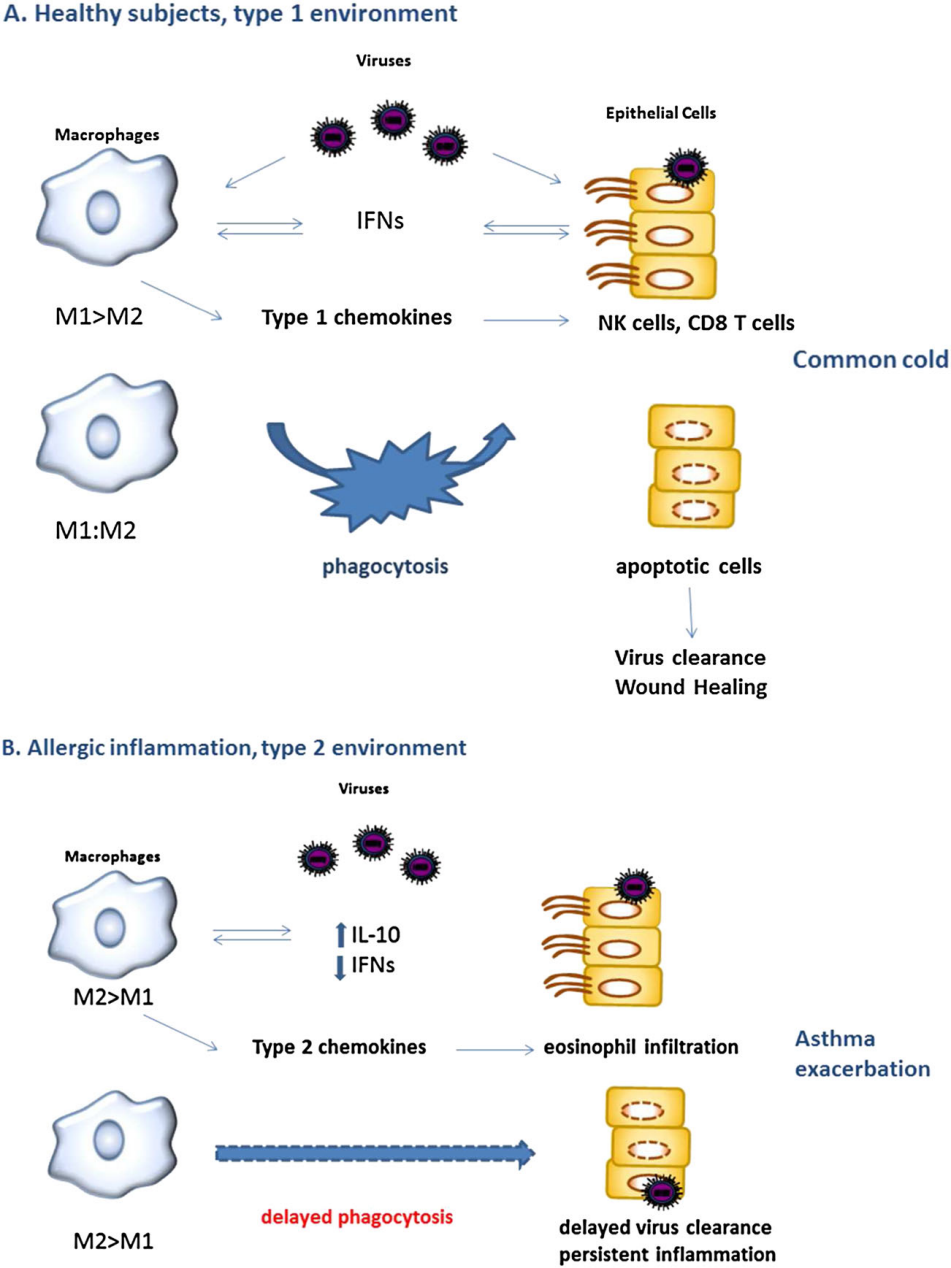
Role of lung MØ in the immune response to respiratory viruses in healthy and asthmatic subjects

Rhinovirus infections cause the majority of asthma exacerbations in adults and children [3,4,131]. It was suggested that IL-10, when already present during a virus infection in a disease characterized by a type 2 cytokine milieu such as allergic asthma, by suppressing antiviral immune responses, could play an important role in RV-induced asthma [132]. MØs, when alternatively activated, are a source of IL-10, which could differentially modulate T-cell responses to RV in human atopic asthmatic subjects, resulting in preferential development of pro-allergic type 2 versus antiviral type 1 T cells. Consequently, the antiviral immune response in atopic asthmatic subjects is deficient during an RV infection at both innate (decreased antiviral capacity of MØs) and adaptive (decreased number/function of antiviral type 1 T cells) immunity levels. All these effects will impair virus eradication and contribute to asthma exacerbation severity and, in the long term too, perhaps airway fibrosis (Figure2).

However, currently, there is limited information on the role of suppressor IL-10 in the anti-inflammatory feedback on innate antiviral immune response in the lung in virus-induced asthma.

Concerning the immune responses to respiratory viruses in a type 2 milieu in animal models of asthma, a recent study showed that MØs from OVA-treated mice expressed eotaxin-1/CCL11, IL-4, and IL-13 in response to RV1B infection *ex vivo*, as well as the MØ alternative activation markers arginase-1, chitinase-like 3, MØ galactose-type C-type lectin 2, and IL-10 [133]. In cultured MØs, ultraviolet irradiation of RV1B abrogated the eotaxin, IL-10, and IFN responses, indicating that RV causes a replicative infection in MØs, and cytokine expression is dependent on viral replication [133]. M2 MØs induced during RV1B infection also exacerbate eosinophilic airway inflammation by producing the chemokine eotaxin 1/CCL11, which recruits eosinophils [133]. Interestingly, RV copy number was decreased in mice with allergic airway diseases, demonstrating an uncoupling of viral load and airway inflammation [133].

Although RVs cause a limited replicative infection in human monocytes/MØs *in vitro*, RV replication modulates surface costimulatory molecule expression and soluble mediators such as IL-10 and cytokine and chemokine production (TNF-α, IL-8/CXCL8, monocyte chemo-attractant protein-1/CCL2, IFN-γ-inducible protein 10 (IP-10)/CXCL10, IL-12, IL-15) and type I and III IFN production [134–145]. *In vitro* RV infection of PBMCs from atopic asthmatic donors shows a shift toward a type 2 phenotype, with increased IL-10 and decreased IFN-γ and IL-12, as compared with normal volunteers, and RV inactivation decreased IL-10 levels, suggesting that IL-10 production was mostly induced by RV replication in monocytes [138]. RV16 increased production of IL-10 in MDMs and inoculum from which virus had been filtered, and soluble ICAM-1-coated RV16 and ultraviolet-inactivated RV16 did not induce IL-10 production, confirming that RV16 replication induces IL-10 production (not virus receptor binding, virus phagocytosis, or soluble factors in the virus inoculum) (presented at 12th International Congress of Immunology July 18–23, 2004, in Montréal, Canada “IL-4 Increases IL-10 Production by RV-Infected MØs,” Stanciu LA, Laza-Stanca, Johnston SL, Poster 814PM, M36.115). Microarray examination of PBMCs from children with acute exacerbations of asthma revealed a comprehensive list of 52 aaMØ signature genes, including CD163, SOCS1, IL-10, and IL-13R [146].

We found that, similar to epithelial cells, *in vitro* MDMs and bronchoalveolar MØs could be infected by RV [141,142], and RV infection of monocytes/MØs induces innate IFN (IFN-α/β, IFN-λ) production in the following: (i) monocytes (PBMCs IFN-α > IFN-β > IFN-λ) [142,144]; and (ii) MDMs (IFN-α/β/λ) and alveolar MØs (IFN-α and IFN-λ) [142,145]. However, we reported that RV infection induces less antiviral IFN-β and IFN-λ in respiratory epithelial cells and less IFN-α/β and IFN-λ in bronchoalveolar cells from atopic asthmatic as compared with normal subjects [142,145,147]. Innate IFN deficiency was correlated with severity of RV16-induced asthma exacerbation and virus load in RV-infected asthmatic volunteers [142,145,147]. We also reported that bronchoalveolar lavage cells from atopic asthmatic subjects show a deficient IL-10 response to lipoprotein that correlated with increased viral load, lower airway inflammation, and lung function impairment in response to experimental RV infection [148].

Subjects with virus-induced acute asthma had significantly higher induced sputum IL-10 as compared with nonviral, acute asthma [149,150]. Higher frequency of M2/aaMØ has been reported during asthma attacks [124,146]. In a study of subjects with acute asthma, stable asthma, and healthy controls, induced sputum gene expression for TLR3, IP-10/CXCL10, and IL-10 was increased in viral, compared with nonviral, acute asthma, and levels of IP-10/CXCL10 and IL-10 mRNA expression were correlated with the mRNA expression of TLR3 in viral acute asthma [150]. However, both stable and acute asthmatic subjects were on inhaled corticosteroid treatment, acute at higher doses compared with the stable asthma group, and there were a mixture of atopic and nonatopic in all three groups.

## Conclusions

In normal subjects, the antiviral immune response via innate IFNs is accompanied by an anti-inflammatory immune response with eradication of the virus. Within this process, IL-10, secreted by activated MØs, induces regulatory T cells and facilitates the resolution of inflammation (Table2).

**Table 2:** Macrophage characteristics during a respiratory virus infection

Macrophages		
Before virus infection	During acute virus infection	Convalescence
Normal subjects		
Cytokines: type 1 > type 2	M1 > M2 functions	Eradication of the virus
Constitutive antiviral innate IFNs	Increased innate IFNs	
M1 > M2 functions	Increased ability to kill	
	Efficient APC	
	High levels of pro-inflammatory cytokines	
	Low levels of virus	
	*Common cold*	
Asthmatic subjects		
Allergic inflammation	M2 > M1 functions	Delayed virus eradication
Cytokines: type 2 > type 1	High levels IL-10	
Excess IL-4	Decreased IFNs production	
	Decreased ability to kill	
M2 > M1 functions	Poor APC	Excessive and prolonged inflammatory responses
	Higher levels of virus	
	*Acute exacerbation of asthma*	

In healthy, normal subjects, respiratory viruses infect resident macrophages, which will become activated (innate activation), produce pro-inflammatory cytokines and antiviral IFN-α/β/λ, which modulate other components of the antiviral immune response, including NK and T cells. IFN-γ produced by NK and T cells in turn modulates MØ function (classical activation). MØ infection also upregulates the antigen-presenting molecules (MHC class I and II and costimulatory molecules). Later during viral infection, MØ phagocytose apoptotic/necrotic cells and participate in the maintenance of effector and memory CD8 T cells, through IL-15 production, helping the further clearing of virus or virus-induced pathology. When viral infection is cleared, MØ undergoes deactivation/alternative activation and participate in reduction of inflammation and healing processes through secreting anti-inflammatory molecules such as IL-10. All these activities are downregulated and delayed in asthma. Reworking after: Laza-Stanca V, Stanciu LA, Johnston SL. *I*n vitro models of macrophage infection. In: *Asthma exacerbations*. Johnston SL & O'Byrne PM (eds). Informa UK Ltd. 2007:223–242*.*

However, when IL-10 levels are already upregulated prior to viral infection such as in allergic asthma, the antiviral immune response is deficient, suggesting that the interaction between IL-10 and innate IFNs is not an “on-and-off” process.

The balance between innate IFNs and IL-10 seems to be a prerequisite for an efficient crosstalk between the innate and adaptive arms of the immune system during viral infections, and it appears to be dysregulated in asthmatic lungs. This is something that needs to be explored further, especially when IL-10 is suggested as a potential therapy in allergy and asthma. More studies investigating these interactions during virus-induced exacerbations of asthma are eagerly anticipated in order to shed more light on the pathogenesis of asthma exacerbations.

## Abbreviations

aaMØ: alternatively activated macrophages
AHR: airway hyperresponsiveness
ARG1: arginase 1
BAL: bronchoalveolar lavage
BN: Brown Norway
caMØ: classically activated macrophages
GATA3: GATA-binding protein 3
GM-CSF: granulocyte macrophage colony-stimulating factor
IFNR: IFN receptor
IL-10R: IL-10 hetero-dimeric receptor
IRF: IFN regulatory factor
ISGF3: IFN-stimulated gene factor 3
LPS: lipopolysaccharide
M1: macrophages, the pro-inflammatory
M2: macrophages, the anti-inflammatory
MDMs: monocyte-derived macrophages
MØs: macrophages
NF-κB: nuclear factor-kappa B
OVA: ovalbumin
pDC: plasmacytoid dendritic cells
RelA: NF-κB subunit p65
RELMα: resistin-like molecule-α
RV: rhinovirus
SD: Sprague Dawley
SOCS3: suppressor of cytokine signaling 3
STAT: signal transducer and activator of transcription
TGF-β: transforming growth factor-beta
TIMPs: tissue inhibitors of metalloproteinases
TIR: Toll/IL-1 receptor
